# Investigating the Association of Patient Body Mass Index With Posterior Subcutaneous Fat Thickness in the Cervical Spine: A Retrospective Radiographic Study

**DOI:** 10.7759/cureus.34739

**Published:** 2023-02-07

**Authors:** Joshua D Piche, Bridger Rodoni, Aditya Muralidharan, Daniel Yang, Joel Gagnier, Rakesh Patel, Ilyas Aleem

**Affiliations:** 1 Department of Orthopaedic Surgery, University of Michigan, Ann Arbor, USA

**Keywords:** preoperative planning, outcomes, complications, surgical site infection, body mass index, subcutaneous fat thickness, cervical spine surgery

## Abstract

Introduction: Although BMI is often used as a surrogate for posterior cervical subcutaneous fat thickness (SFT), the association of BMI with cervical SFT is unknown. We performed a retrospective radiographic study to analyze the relationship between BMI and cervical SFT.

Methods: This was a retrospective cohort study of patients with cervical CT scans. SFT was assessed by measuring the distance (mm) from the spinous processes of C2-C7 to the skin edge. Pearson correlations and linear regression were used to analyze the relationship between BMI and SFT. One-way ANOVA was used to analyze differences in C2-C7 distances while stratifying by BMI.

Results: A total of 96 patients were included. BMI had a moderate correlation with average C2-C7 (*r*=0.546, p < 0.05) SFT*, *and a weak to moderate correlation with each individual C2-C7 distance. The strongest correlation was at the C7 level (*r*= 0.583, p < 0.05). These analyses remained significant controlling for potential confounders of patient age, sex, and diabetes. No difference was found in the average C2-C7 distance in patients with BMIs of 25-30 compared to those with BMIs of 30-40 (p=0.996), whereas in patients with BMI <25 and BMI >40, differences were significant (p < 0.05).

Conclusions: BMI is not strongly correlated with SFT in the cervical spine. Although BMI less than 25 or greater than 40 is correlated with respectively decreased or increased cervical SFT, BMI of 25-40 is not correlated with cervical SFT. This is clinically important information for surgeons counseling patients on perioperative risk before undergoing cervical spine procedures, namely infection. Further research delineating the relationship between posterior SFT and surgical site infection in the cervical spine is warranted.

## Introduction

Surgical site infection (SSI) is a known complication following cervical spine surgery, with rates as high 0.07% after anterior cervical surgery [1] and up to 18% after posterior surgery [2,3]. Risk factors for SSI include increased BMI, truncal obesity, smoking, diabetes, longer operative times, and a history of prior SSI [4,5]. Obesity and BMI have gained much attention in regard to preoperative planning and counseling patients, especially as the rates of obesity continue to rise in the United States. The Centers for Disease Control (CDC) defines obesity as a BMI of 30 or higher, and severe obesity as a BMI of 40 or higher. The prevalence of obesity in 2017-2018 was 42.4%, as compared to 30.5% in 1999-2000. Similarly, the rate of severe obesity increased from 4.7% to 9.2% in that same time period [6]. A BMI of 30 or above was shown to have an increased risk of postoperative infection in patients undergoing spine surgery, with an odds ratio of 2.13 [5]. The increasing prevalence of obesity and known complications intraoperatively and postoperatively have led to the advocacy for diligent preoperative counseling and optimization on the part of the spine surgeon [7,8]. Diet and exercise counseling, as well as referral to bariatric surgeons when appropriate, should all be considered to help optimize surgical outcomes in elective spine procedures for obese patients [7,8].

Although patient BMI is often used preoperatively to assess the risk for postoperative infection, it must be recognized that this has potential limitations in certain patients. BMI is simply a function of a patient’s height and weight, and because of this, it cannot reliably measure regional fat distribution in the operative area of interest [9,10]. This is important in spinal surgery because while a patient may have a high BMI, this could be due to truncal obesity, and may not necessarily reflect the amount of adipose tissue at their spinal surgical site. This realization has led to the postulation that local subcutaneous fat thickness (SFT) may be a better predictor of SSI than BMI. In the lumbar spine, BMI and obesity were not significantly related to SSI, but rather, SFT did show a significant relationship, with a 6% increase in odds of infection for every 1-mm increase in local SFT [9]. BMI and fat thickness were found to be only weakly correlated (r2=0.44) in the lumbar spine [9]. In a previous study assessing patients who underwent posterior cervical fusion (PSF), it was shown that BMI and obesity were not significant risk factors for postoperative infection, but increased SFT at the C5 level was [11].

To our knowledge, the association between patient BMI and the thickness of the posterior cervical subcutaneous fat has not been previously reported. The limited literature on this topic leaves a void in knowledge on whether or not BMI can be used as a reliable surrogate marker for local subcutaneous fat when counseling patients preoperatively on the potential risks of undergoing a cervical spine procedure. We performed a retrospective cohort imaging review study to test the hypothesis that patient BMI is not strongly correlated with local SFT throughout the cervical spine.

## Materials and methods

Study design and inclusion/exclusion criteria

This retrospective cohort study was granted an exemption by the Institutional Review Board at the University of Michigan, Ann Arbor, Michigan, United States. A manual electronic chart review of the senior author's (IA) clinic patients from January 1, 2018, to December 31, 2019, was used to identify patients. Patients were eligible if they were at least 18 years old and presenting to the spine clinic with a non-contrast CT scan of the cervical spine. Additional inclusion criteria used were: CT scans had to be preoperative, and a BMI or height and weight within three months of the CT scan had to be recorded in the patient chart. Patients were excluded if they had previous cervical spine surgery, if there was significant coronal or sagittal cervical deformity present, or if the CT scan did not allow adequate visualization and measurement of all pertinent levels for this study.

Outcomes and measurements

**Figure 1:**
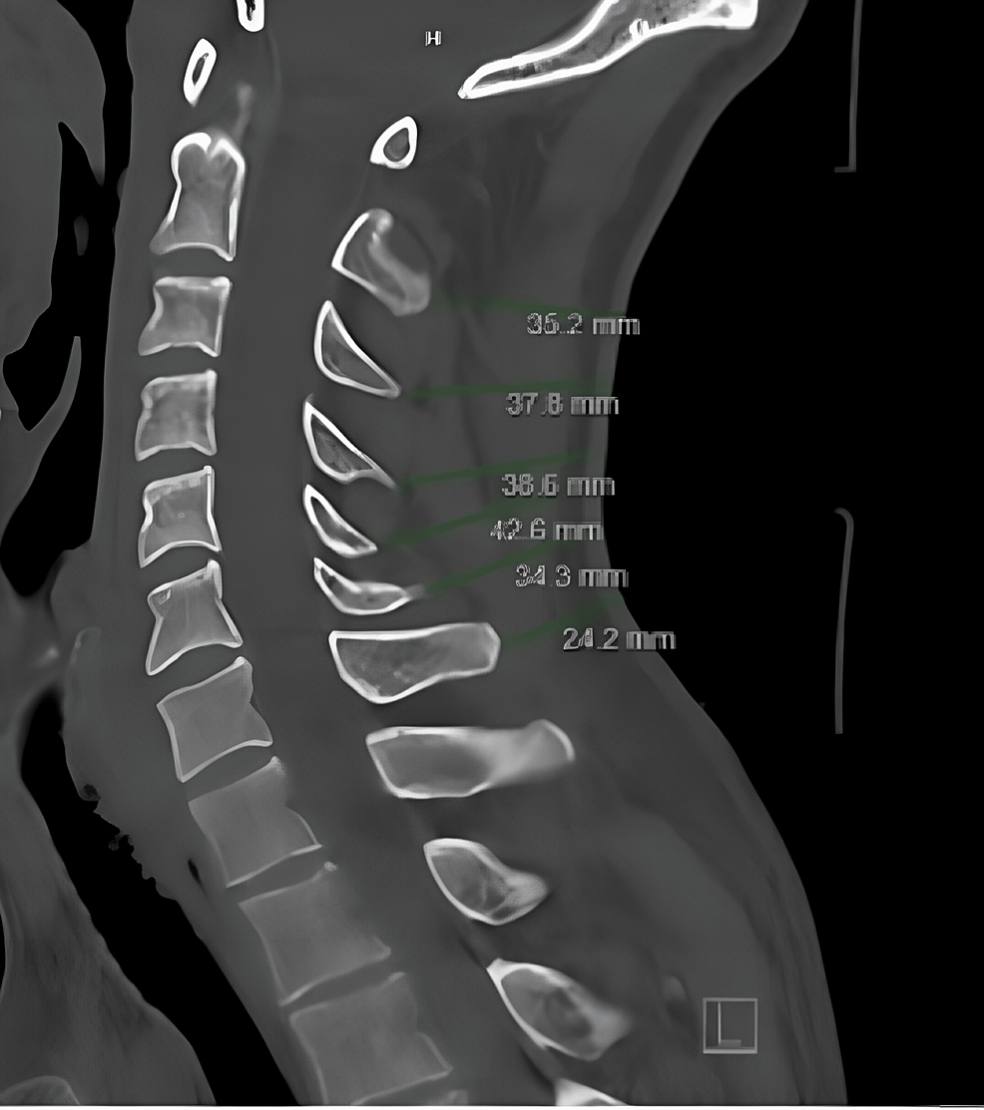
Representative sagittal cervical CT scan showing standard C2-C7 measurements

**Figure 2:**
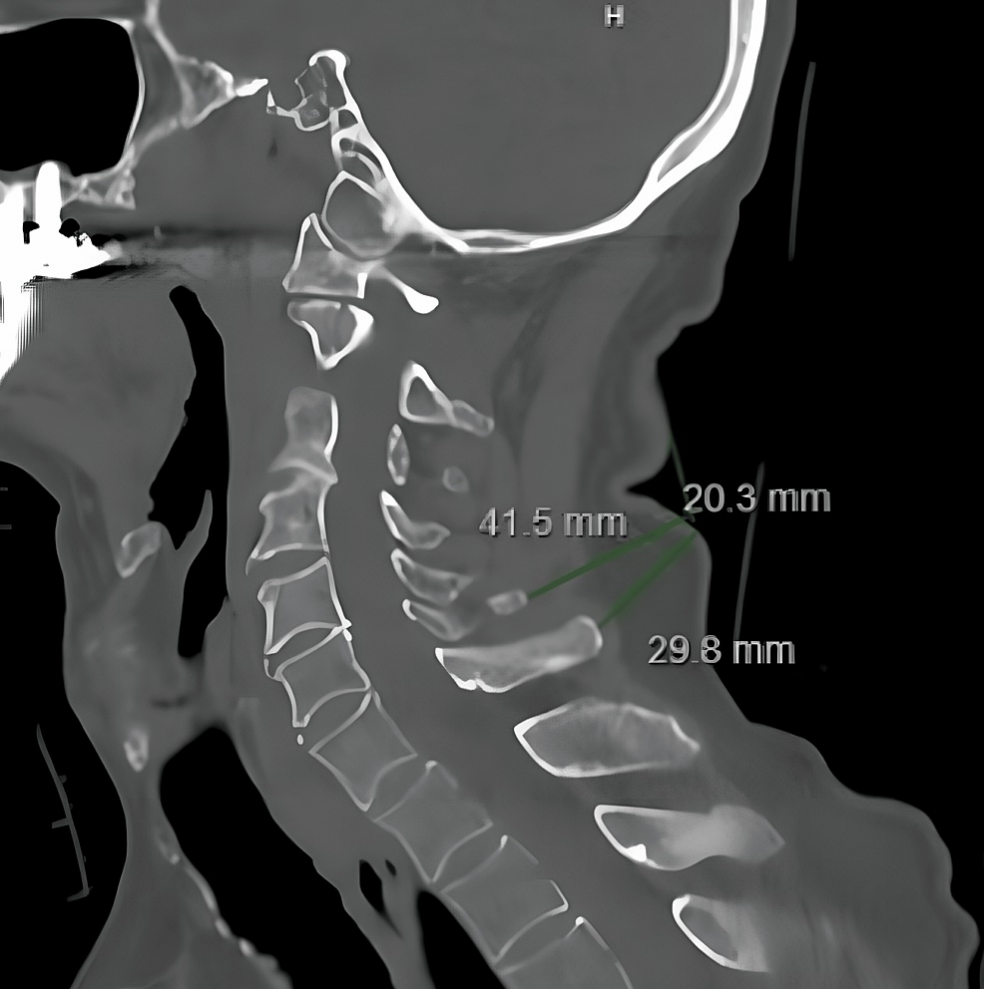
Representative sagittal cervical CT scan showing measurements in a patient with a large posterior skin fold

Electronic medical charts were reviewed to collect demographic patient data including age, sex, BMI, and presence of diabetes. A single study member (JP) then performed all relevant measurements of the CT scans for consistency. The measurements obtained included the distance (mm) from the spinous processes of the C2-C7 levels to the skin edge, measured parallel to the disc space at the respective level on sagittal images (Figure 1). For patients with large posterior skin folds, the measurements at these levels were obtained by extending out to a line that connected the skin above and below the fold, in order to help eliminate any artificial inaccuracy due to patient anatomy or position during the CT (Figure 2). Each measurement was saved and reviewed by two additional study members (JP, IA) to ensure accuracy and consistency. Any discrepancy in measurement warranted a re-measurement of that specific patient.

Statistical analyses

Using an alpha of 0.05 and power of 80%, we calculated an estimated sample size of 50 patients. To improve the precision of the results and allow for subgroup analyses, we exceeded this sample size. Statistical analyses were performed using IBM SPSS Statistics for Macintosh, Version 26.0 (Released 2019; IBM Corp., Armonk, New York, United States) and Stata Statistical Software: Release 14/MP (2015; StataCorp LP, College Station, Texas, United States).

Bivariate Pearson’s correlation analyses were used to determine the correlation between BMI and posterior SFT at individual levels from C2-C7, as well as between BMI and the average total C2-C7 distances. Correlations were stratified with r = 0.30 to 0.50 indicating a weak relationship, r = 0.50 to 0.70 indicating a moderate relationship, and r > 0.70 indicating a strong relationship [12,13]. These same analyses were then put in a linear regression model controlling for patient age, gender, and the presence of diabetes. One-way ANOVAs were performed to assess for differences in average C2-C7 distances while stratifying by BMI groups of less than 25 (normal), 25 to less than 30 (overweight), 30 to less than 40 (obese), and greater than 40 (morbid obesity). A p < 0.05 was used to determine the statistical significance for all tests.

## Results

A total number of 96 patients with a mean age of 62.0 years (± 17.7) were included in the final analysis, with 45.8% (n=44) being female. The average patient BMI was 28.2 (±6.3). Patient demographics are shown in Table 1. The average distances (mm) at each individual level from C2-C7 to the skin edge for the cohort were as follows: C2 44.6 (±12.8), C3 45.9 (±12.9), C4 44.4 (±13.7), C5 41.5 (±13.8), C6 35.7 (±12.3), and C7 28.7 (±10.9).

**Table 1:** Participant demographics

Age (years), mean (SD)		62.0 (17.7)
Gender, N (%)	Male	52 (54.2%)
	Female	44 (45.8%)
BMI, mean (SD)		28.2 (6.3)
BMI Range		17.99-50.15
Diabetes, N (%)	Yes	24 (25%)
	No	72 (75%)

**Figure 3:**
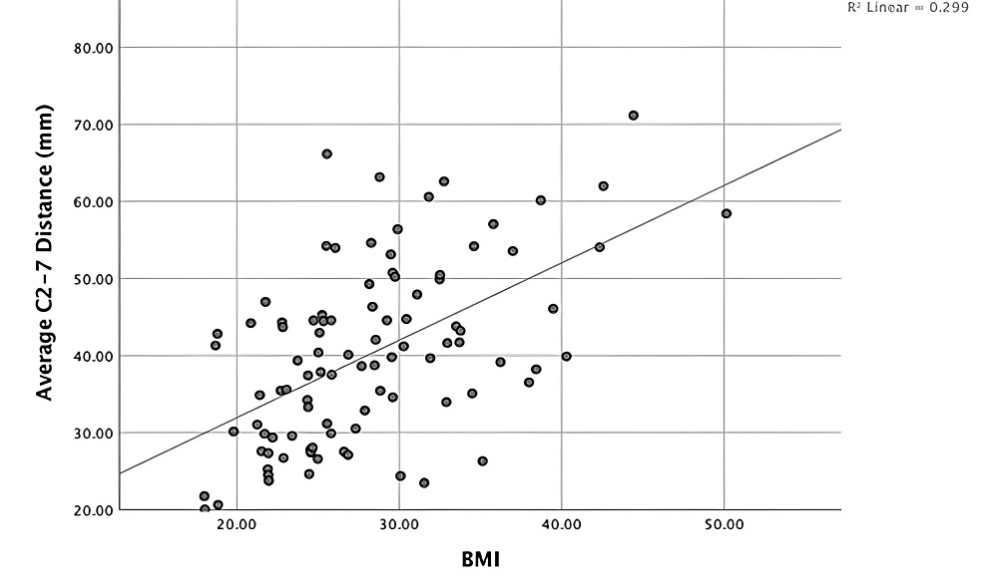
Graphical representation of the association between patient BMI and the average C2-C7 subcutaneous fat thickness

The total average distance (mm) from the C2-C7 spinous processes to the skin edge was found to be 40.1 (±11.6). When analyzing the relationship between the average total C2-C7 distance and patient BMI using bivariate Pearson correlations, a moderate relationship was found (r = 0.546, p = <0.05) (Figure 3). This relationship also remained significant using a linear regression model controlling for patient age, sex, and the presence of diabetes (p < 0.05), with an R2 of 0.421.

BMI had a weak to moderate correlation with SFT at each individual level of C2-C7. The strongest bivariate Pearson correlation was seen at the C7 level (r=0.583). The remaining levels showed correlations of: C2 (r=0.511), C3 (r=0.481), C4 (r=0.473), C5 (r=0.439), and C6 (r=0.508). All p-values were significant at <0.05. Linear regression models controlling for patient age, sex, and the presence of diabetes remained significant at all levels (p= <0.05).

**Figure 4:**
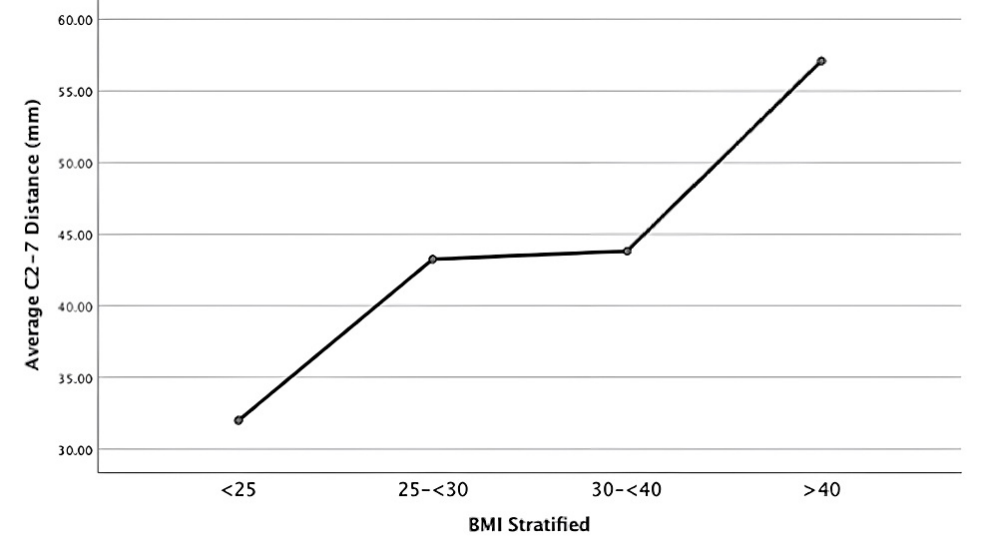
Graphical representations of the average C2-C7 subcutaneous fat thickness stratified by BMI cohort

Patients were stratified into four groups based on BMI: Group 1= BMI <25, Group 2= BMI 25 to <30, Group 3= BMI 30 to <40, and Group 4=BMI>40. Group 1 (n = 34) had an average C2-C7 SFT of 31.9 mm (±7.7), while those in Group 2 (n=32) had an average C2-C7 SFT of 43.3 mm (±9.9) (p< 0.05). Patients in Group 3 (n=25) had an average C2-C7 SFT of 43.8 mm (±10.8). The average C2-C7 SFT of those in Group 4 (N=5) was 57.1 mm (±11.5). One-way ANOVA analyses showed that the average total C2-C7 SFT was significantly lower in Group 1 compared to the other three BMI groups. Similarly, Group 4 had a significantly higher C2-C7 average SFT compared to the other three groups. However, those in groups 2 and 3 were not significantly different from each other (p=0.996). See Table 2 and Figure 4 for further details.

**Table 2:** One-way ANOVA analyses of C2-C7 average subcutaneous fat thickness compared to stratified BMI

Comparison BMI Group	Subcutaneous Fat Thickness Difference (Mean)	P-value	95% Confidence Interval
1 vs 2	-11.3	<0.0005	(-17.4 to -5.1)
1 vs 3	-11.8	<0.0005	(-18.4 to -5.3)
1 vs 4	-25.1	<0.0005	(-37.0 to -13.2)
2 vs 3	-0.56	0.996	(-7.2 to 6.1)
2 vs 4	-13.8	0.017	(-25.8 to -1.9)
3 vs 4	-13.3	0.028	(-25.5 to -1.1)

## Discussion

Our data indicate that BMI did not have a strong correlation with cervical SFT. When stratifying the cohort into BMI groups of <25, 25 to <30, 30 to <40, and >40, it was shown that the weak-moderate correlation that was found is likely driven by those at the extremes of BMI. This is demonstrated by the fact that those with BMI<25 or BMI>40 did have significantly different SFT compared to the other groups; however, those with a BMI of 25 to <30 and 30 to <40, were not significantly different from each other. Clinically, the results as a whole suggest that solely using patient BMI as a surrogate for local SFT and SSI risk is not reliable. This is especially true for those at the norm with BMIs of 25-40, who showed no significant differences in their SFT.

Preoperative identification of risk factors that increase the potential for perioperative complications is an essential component of surgical decision-making. Understanding these risks and how to potentially minimize them are critical to shared decision-making with the patient. While the absolute percentages of SSIs after cervical spine surgery are relatively low [1,2,14,15], the effect on patient outcomes and the financial impact on the health system, are certainly not inconsequential. A retrospective study of patients with SSI after spine surgery showed an average secondary readmission stay of 9.6 days and an average cost of treatment of $19,642 USD [3]. A systematic review showed a mortality rate attributable to spine SSI to range from 1.1% to 2.3%, and also showed that spine patients incur double the healthcare costs when they develop a postoperative infection [16].

Although BMI has previously been shown to be a risk factor for SSI, recent studies have challenged this and shown that local SFT in the lumbar and cervical spine, rather than BMI, is actually a better predictor of SSI [9,11]. Local SFT, however, is not a routinely obtained measurement. Instead, BMI is often used as a surrogate measure due to its perceived correlation with SFT and ease of use in registry-based studies. However, no prior study to our knowledge has directly investigated the relationship between BMI and local SFT in the cervical spine. In the lumbar spine, Lee et al. showed weak correlations (r=0.41-0.49) between BMI and local SFT [9]. In the cervical spine, Mehta et al. reported a weak correlation (r=0.23) between BMI and SFT at the C5 level, but no other cervical levels were analyzed, and this was not the primary outcome of interest in their study [11]. The results of the present study are in concordance with prior studies in the lumbar spine demonstrating a correlation that was not strong between BMI and local SFT. At best, the correlation was moderate, primarily at the C7 level.

Strengths and limitations

The strengths of this study include the relatively large cohort, consistent CT-based measurements reviewed by three authors, and the fact that this is the first study to directly investigate the relationship between BMI and local posterior cervical SFT. Limitations include the fact that this was a single-center and single-surgeon experience, as well as the retrospective nature of the study. Further, it should be clear that the present study does not investigate the relationship between BMI or SFT and SSI. It is possible that SFT may be more strongly correlated with postoperative SSI than BMI, but this hypothesis requires further investigation. In theory, in any future effect that is observed, increased posterior SFT should only have a direct impact on infection in posterior cervical cases. However, we also plan to include anterior cervical procedures in future studies.

## Conclusions

Patient BMI is not strongly correlated with posterior SFT in the cervical spine. Although BMI less than 25 or greater than 40 is correlated with respectively decreased or increased cervical SFT, BMI of 25-40 did not show a significant correlation with cervical SFT. This is clinically important information for surgeons counseling patients on perioperative risk before undergoing cervical spine procedures, namely infection. In theory, increased posterior SFT can place patients at higher risk of infection after posterior cervical spine surgery, independent of patient BMI. This factor is not actively considered by most surgeons during the preoperative evaluation. Further research delineating the relationship between posterior SFT and SSI in the cervical spine is warranted.

## References

[1] Ghobrial GM, Harrop JS, Sasso RC (2017). Anterior cervical infection: presentation and incidence of an uncommon postoperative complication. Global Spine J.

[2] Aleem IS, Tan LA, Nassr A, Riew KD (2020). Infection prevention in cervical spine surgery. J Spine Surg.

[3] Barnes M, Liew S (2012). The incidence of infection after posterior cervical spine surgery: a 10 year review. Global Spine J.

[4] Xing D, Ma JX, Ma XL (2013). A methodological, systematic review of evidence-based independent risk factors for surgical site infections after spinal surgery. Eur Spine J.

[5] Meng F, Cao J, Meng X (2015). Risk factors for surgical site infections following spinal surgery. J Clin Neurosci.

[6] Hales CM, Carroll MD, Fryar CD, Ogden CL (2020). Prevalence of obesity and severe obesity among adults: United States, 2017-2018. NCHS Data Brief.

[7] Spina NT, Aleem IS, Nassr A, Lawrence BD (2018). Surgical site infections in spine surgery: preoperative prevention strategies to minimize risk. Global Spine J.

[8] Aleem IS, Tan LA, Nassr A, Riew KD (2020). Surgical site infection prevention following spine surgery. Global Spine J.

[9] Lee JJ, Odeh KI, Holcombe SA, Patel RD, Wang SC, Goulet JA, Graziano GP (2016). Fat thickness as a risk factor for infection in lumbar spine surgery. Orthopedics.

[10] Kato MM, Currier MB, Villaverde O, Gonzalez-Blanco M (2005). The relation between body fat distribution and cardiovascular risk factors in patients with schizophrenia: a cross-sectional pilot study. Prim Care Companion J Clin Psychiatry.

[11] Mehta AI, Babu R, Sharma R (2013). Thickness of subcutaneous fat as a risk factor for infection in cervical spine fusion surgery. J Bone Joint Surg Am.

[12] Mukaka M (2012). A guide to appropriate use of Correlation coefficient in medical research. Malawi Med J.

[13] Landis JR, Koch GG (1977). The measurement of observer agreement for categorical data. Biometrics.

[14] Sebastian A, Huddleston P 3rd, Kakar S, Habermann E, Wagie A, Nassr A (2016). Risk factors for surgical site infection after posterior cervical spine surgery: an analysis of 5,441 patients from the ACS NSQIP 2005-2012. Spine J.

[15] Blumberg TJ, Woelber E, Bellabarba C, Bransford R, Spina N (2018). Predictors of increased cost and length of stay in the treatment of postoperative spine surgical site infection. Spine J.

[16] Patel H, Khoury H, Girgenti D, Welner S, Yu H (2017). Burden of surgical site infections associated with select spine operations and involvement of staphylococcus aureus. Surg Infect (Larchmt).

